# Genetic Testing and Its Clinical Application in Prostate Cancer Management: Consensus Statements from the Hong Kong Urological Association and Hong Kong Society of Uro-Oncology

**DOI:** 10.3389/fonc.2022.962958

**Published:** 2022-07-18

**Authors:** Peter K. F. Chiu, Eric K. C. Lee, Marco T. Y. Chan, Wilson H. C. Chan, M. H. Cheung, Martin H. C. Lam, Edmond S. K. Ma, Darren M. C. Poon

**Affiliations:** ^1^ S.H. Ho Urology Centre, Department of Surgery, The Chinese University of Hong Kong, Hong Kong SAR, China; ^2^ Department of Clinical Oncology, Tuen Mun Hospital, Hong Kong SAR, China; ^3^ Division of Urology, Department of Surgery, Tuen Mun Hospital, Hong Kong SAR, China; ^4^ Division of Urology, Department of Surgery, United Christian Hospital, Hong Kong SAR, China; ^5^ Division of Urology, Department of Surgery, Tseung Kwan O Hospital, Hong Kong SAR, China; ^6^ Department of Oncology, United Christian Hospital, Hong Kong SAR, China; ^7^ Department of Pathology, Hong Kong Sanatorium and Hospital, Hong Kong SAR, China; ^8^ Department of Clinical Oncology, State Key Laboratory of Translational Oncology, Sir YK Pao Centre for Cancer, Hong Kong Cancer Institute, The Chinese University of Hong Kong, Hong Kong SAR, China; ^9^ Comprehensive Oncology Centre, Hong Kong Sanatorium and Hospital, Hong Kong SAR, China

**Keywords:** Asians, genetic counseling (MeSH), genetic testing, hereditary cancer syndromes, liquid biopsy, molecular targeted therapy, practice guideline (MeSH), prostate cancer

## Abstract

**Background:**

In recent years, indications for genetic testing in prostate cancer (PC) have expanded from patients with a family history of prostate and/or related cancers to those with advanced castration-resistant disease, and even to early PC patients for determination of the appropriateness of active surveillance. The current consensus aims to provide guidance to urologists, oncologists and pathologists working with Asian PC patients on who and what to test for in selected populations.

**Methods:**

A joint consensus panel from the Hong Kong Urological Association and Hong Kong Society of Uro-Oncology was convened over a series of 5 physical and virtual meetings. A background literature search on genetic testing in PC was performed in PubMed, ClinicalKey, EBSCOHost, Ovid and ProQuest, and three working subgroups were formed to review and present the relevant evidence. Meeting agendas adopted a modified Delphi approach to ensure that discussions proceed in a structured, iterative and balanced manner, which was followed by an anonymous voting on candidate statements. Of 5 available answer options, a consensus statement was accepted if ≥ 75% of the panelists chose “Accept Completely” (Option A) or “Accept with Some Reservation” (Option B).

**Results:**

The consensus was structured into three parts: indications for testing, testing methods, and therapeutic implications. A list of 35 candidate statements were developed, of which 31 were accepted. The statements addressed questions on the application of PC genetic testing data and guidelines to Asian patients, including patient selection for germline testing, selection of gene panel and tissue sample, provision of genetic counseling, and use of novel systemic treatments in metastatic castration-resistant PC patients.

**Conclusion:**

This consensus provides guidance to urologists, oncologists and pathologists working with Asian patients on indications for genetic testing, testing methods and technical considerations, and associated therapeutic implications.

## 1 Introduction

The incidence of prostate cancer (PC) has risen rapidly in Asia (1). In Hong Kong, PC was the third most commonly diagnosed male cancer in 2019, with the highest rate of increase in new cases among all cancers from the previous year (2).

Although genetic testing has traditionally not been performed in PC patients, there is growing evidence for its role in providing additional prognostic and therapeutic information for selected patient populations. Sequencing studies demonstrated that ~11.8% of metastatic PC (mPC) patients carry inherited DNA repair gene mutations based on germline testing (3), and ~23% of castration-resistant prostate cancer (CRPC) patients show DNA repair pathway aberrations on somatic testing (4). Currently, poly (adenosine diphosphate-ribose) polymerase inhibitor (PARPi) treatment is indicated for metastatic CRPC (mCRPC) patients who harbor homologous recombination repair (HRR) gene mutations, including *BRCA1/2* or *ATM*, which showed an overall survival (OS) benefit in the PROfound trial (5, 6).

In recent years, indications for genetic testing in PC have expanded from patients with a family history (FH) of prostate and/or related cancers to those with advanced castration-resistant disease, and even to early PC patients for determination of the appropriateness of active surveillance (AS) (7). The latest international guidelines and consensuses provide guidance and perspectives on the indications and modes of genetic testing in PC, including relevant clinical scenarios where patient management could be affected by the identification of a pathogenic mutation. Depending on the indication for genetic testing, the selection of testing method and technique will likely vary, such as germline versus somatic testing, or tumor versus blood sampling. For somatic testing, when tumor tissue is not readily available, circulating tumor DNA (ctDNA) from blood plasma may provide a convenient alternative (8, 9).

Despite the availability of data and international guidelines in support of PC genetic testing, formidable barriers exist in applying the latest developments to an Asian setting. First, caution must be exercised in the direct application of Caucasian-based guidelines to Asian men with PC because Asian data for such testing are limited, and inter-ethnic differences in patterns of genetic mutations (10) and pharmacogenomics (11) are notable. Second, the lack of genetic counseling resources for hereditary conditions can deter physicians from suggesting germline testing to patients, even when a positive germline mutation (*e.g.* germline *BRCA* mutation) seems plausible. Third, an optimal testing panel will need to be defined, taking into consideration the given indication and setting.

To facilitate the development of strategies for genetic testing in PC in Hong Kong, an Asian setting, a local expert consensus was jointly organized by the Hong Kong Urological Association (HKUA) and Hong Kong Society of Uro-Oncology (HKSUO), which are the two most representative professional organizations for PC management in Hong Kong. The statements were derived from available literature and overseas guidelines, and supplemented with the expertise of panel members where evidence was limited.

## 2 Methods

### 2.1 Panel Formation and Meetings

The joint consensus panel was formed from 8 local experts: 4 urologists representing the HKUA, 3 oncologists representing the HKSUO, and 1 invited pathologist (**Supplementary Table 1** and **Appendix A**). A series of 5 meetings were held for literature presentations, discussions and voting (**Supplementary Table 2**).

### 2.2 Topics and Literature Review

A literature review of 5 medical databases was conducted to retrieve major publications that were relevant to the present study: PubMed, ClinicalKey, EBSCOHost, Ovid and ProQuest. The basic keywords used were “genetic testing” and “prostate cancer”. Additional keywords were “BRCA”, “castration-resistant”, “chemotherapy”, “Delphi method”, “family history”, “germline”, “guideline”, “homologous recombination repair”, “liquid biopsy”, “metastatic”, “mismatch repair”, “PARP inhibitor” and “somatic.” A list of 47 major articles were selected for in-depth discussion, which comprised 30 large-scale studies, 2 meta-analyses, 6 major guidelines, 6 consensuses and 3 high-impact narrative reviews.

### 2.3 Modified Delphi Method

Where there is incomplete evidence, the Delphi method can be used to make estimations and predictions, determine collective values, and define foundational concepts (12). This method has been employed in recent genitourinary cancer consensuses (13–15). In this study, the following modified Delphi method was used to ensure that the panel discussions would proceed in a structured and iterative manner, contents would be well-balanced, and that participants could contribute fairly and equally: 1) for each of the 3 identified debate areas, a working subgroup was formed to review and present the latest literature evidence for deliberation; 2) each subgroup was also responsible for drafting a set of candidate consensus statements, which were further revised during the discussions, and compiled into the final voting statements; 3) each statement was discussed around the table twice, over two meetings; and 4) at the final meeting, panelists were instructed to vote anonymously on each statement’s “practicability of recommendation” in the locality, based on a set of predefined judgement criteria (**Supplementary Table 3**). A consensus statement was accepted if ≥ 75% of the panelists chose “Accept Completely” (Option A) or “Accept with Some Reservation” (Option B) (16). (**Supplementary Table 4** contains the complete voting record.)

## 3 Results

**Figure 1:**
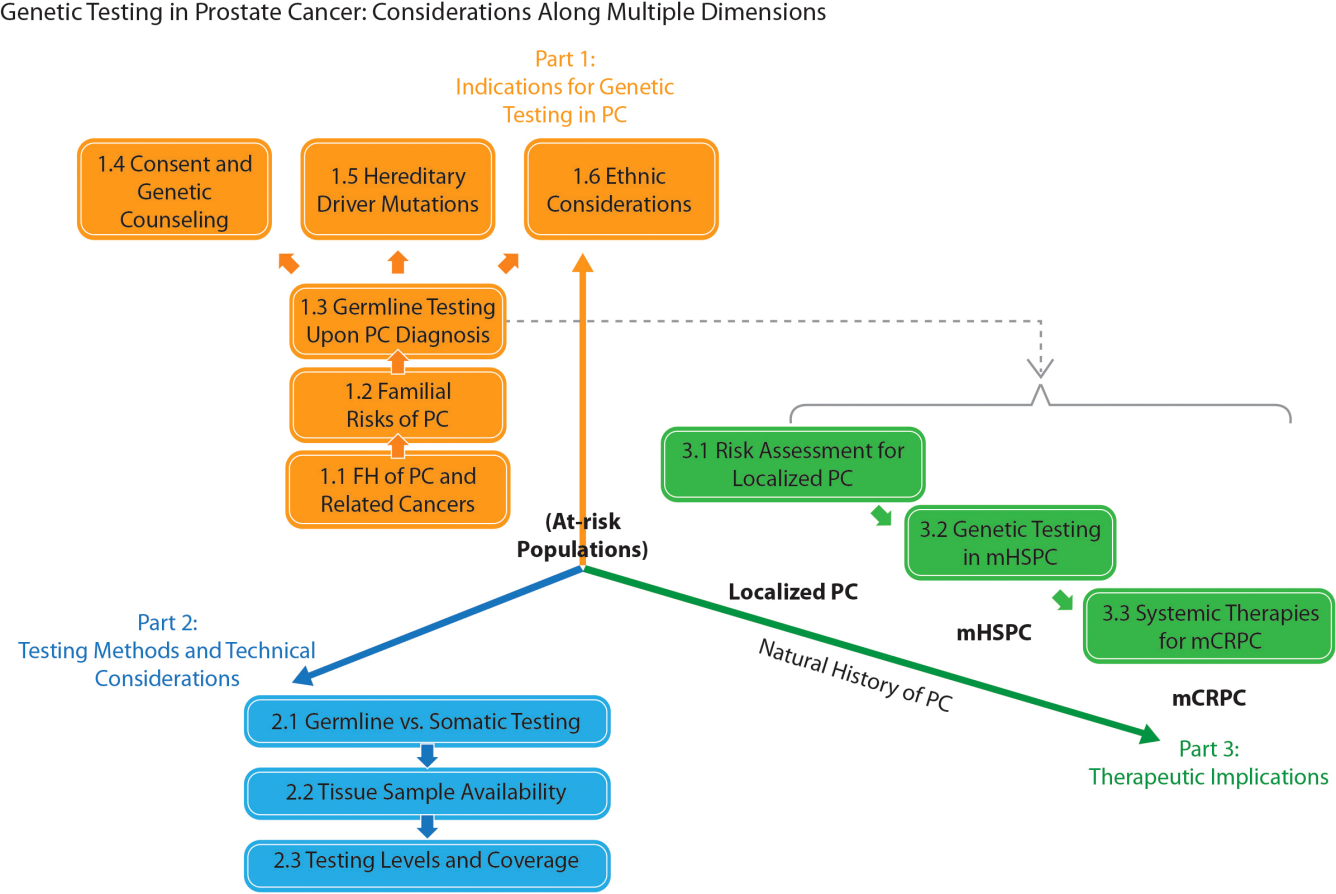
A “patient journey” schematic representation of the topic division for the consensus in prostate cancer (PC) genetic testing. FH, family history; mCRPC / mHSPC, metastatic castration-resistant / hormone-sensitive prostate cancer.

Preliminary discussions converged on a “patient journey” approach to structuring the consensus (**Figure 1**). Moreover, 3 core debate areas were identified: 1) indications for genetic testing in PC; 2) testing methods and technical considerations; and 3) therapeutic implications. A total of 35 candidate statements were finalized for voting. The voting accepted 31 statements (**Table 1**); explanations for their acceptance are given below. **Table 2** provides a brief summary of the consensus, organized by patient disease status. (Voting records, rejected statements, and the latter’s explanations can be found in **Supplementary Tables 4**, **5** and **Appendix B**, respectively.)

**Table 1:** Panel consensus on prostate cancer (PC) genetic testing.

Consensus Statements		Ref.
1. Indications for Genetic Testing in PC		
1.1 FH of PC and Related Cancers		
1.1.1	FH of PC and/or related cancers is defined as having any of: - PC (except localized Grade Group 1) in brother, father or multiple family members diagnosed at age < 60 years; - Death from PC in a first-degree relative aged < 60 years; - Known germline mutations of *BRCA1/2* or DNA MMR genes in the family; - *BRCA1/2m-*associated cancer or Lynch syndrome (namely: bile duct, breast, colorectal, endometrial, gastric, kidney, melanoma, ovarian, pancreatic, prostate [except localized Grade Group 1], small bowel or urothelial cancer) in ≥ 3 members on the same side of the family.	(17–20)
1.2 Familial Risks of PC		
1.2.1	FH of prostate and related cancers should be obtained for patients with newly diagnosed PC.	(19, 21)
1.2.2	Cancer surveillance and prophylactic measures should be discussed with germline mutation carriers.	(19, 22)
1.2.3	For *BRCA2m* carriers, PC screening may start at age 40 years.	(18, 23)
1.2.4	For *BRCA2m* carriers, PC screening may be performed 10 years before the youngest PC diagnosis in the family.	(18)
1.3 Germline Testing Upon PC Diagnosis		
1.3.1	Germline testing should be considered in PC patients with any of the following: - Metastatic disease - Ductal or intraductal histology - Positive FH.	(24–26)
1.4 Consent and Genetic Counseling		
1.4.1	Germline genetic testing should be coupled with informed consent and the provision of genetic counseling for adequate management.	(27)
1.4.2	In Hong Kong, genetic counseling resources are scarce. There is a large unmet need of patients with suspected cancer-associated mutations who would benefit from genetic counseling services from accredited providers.	(28, 29)
1.5 Hereditary Driver Mutations		
1.5.1	HRR genes (*BRCA1/2, ATM, PALB2*) and MMR genes (*MLH1, MSH2, MSH6* and *PMS2*) should be considered in germline testing for PC patients.	(18, 19, 30–32)
1.6. Ethnic Considerations		
1.6.1	In Hong Kong, the knowledge base on genetic testing in PC (indications, choices and implications, etc.) may be quite limited. Genomic research in Hong Kong PC patients (*e.g. BRCAm* prevalence and variants) would help to clarify the local situation.	(33–37)
2. Testing Methods and Technical Considerations		
2.1 Germline *vs*. Somatic Testing		
2.1.1	Genetic variants detected by somatic (or tumor) testing that are potentially inherited, especially those involving the HRR or MMR genes, should be subject to germline confirmation by testing a peripheral blood sample.	(28, 38)
2.1.2	Large genomic rearrangements (LGR), for example exon level deletions of the *BRCA1/2* genes, may escape detection on somatic (tumor) testing by NGS. If FH is positive or the patient is otherwise suspected of inherited cancer, germline genetic testing by a peripheral blood sample should be considered.	(39)
2.2 Tissue Sample Availability		
2.2.1	Formalin-fixed paraffin-embedded (FFPE) tumor tissue submitted for genomic profiling should be examined by a histopathologist to confirm the diagnosis and to identify the region of interest for tumor cell enrichment as indicated for somatic testing.	(40)
2.2.2	The availability and quality of biopsy samples should be considered when somatic testing is planned, since longer tissue storage duration is associated with lower testing success rates.	(41, 42)
2.3 Testing Levels and Coverage		
2.3.1	Genomic profiling should be performed by NGS panel that covers the potentially actionable targets in PC such as DNA damage repair (HRR, MMR and Fanconi anemia genes, and CDK12), phosphatidylinositol-3-kinase (PI3K), and RAS/RAF/MEK pathways.	(43, 44)
2.3.2	Mutational study of the HRR genes is used to indicate homologous recombination defect (HRD). However, HRD is not completely covered by gene mutational study but may need other tests such as genomic signatures or functional assays to detect.	(45, 46)
2.3.3	Apart from direct sequencing of the MMR genes or promoter methylation study, MMR defect is also indicated by MSI phenotype as detectable by PCR on paired tumor normal sample, NGS genomic profiling, or immunohistochemistry (IHC) study of MMR gene expression on tumor cells.	(47, 48)
2.3.4	There are biomarkers for predicting anti-PD-1 effects, *e.g.* tumor mutational burden, MSI and PD-L1 expression by IHC, but the results of these tests may not correlate with one another.	(49, 50)
2.3.5	Apart from tissue biopsy, liquid biopsy for circulating tumor DNA (ctDNA) is an emerging, practical, minimally invasive solution to identify predictive or prognostic genomic alterations and to monitor therapy response, especially in patients with inaccessible tumor or who are poor surgical candidates.	(8, 9)
3. Therapeutic Implications		
3.1. Risk Assessment for Localized PC Patients		
3.1.1	*BRCA2m* carriers who are PC patients should not be offered active surveillance.	(7, 51)
3.1.2	Some tissue-based genetic assays (which are mostly related to cell cycle mutations; *e.g.* Prolaris, Decipher and Oncotype Dx) can provide useful information for detailed risk assessment in localized PC, and consequently, counseling patients on active surveillance or treatment.	(52–57)
3.2 Genetic Testing in mHSPC		
3.2.1	Based on current evidence, genetic testing (including genes involved in DNA HRR, such as *BRCA1/2*) might not have an impact on initial treatment selection in patients with mHSPC, but may be helpful for prognostic counseling and longer-term treatment planning.	(58, 59)
3.3 Systemic Therapies for mCRPC		
Genetic testing can help to guide the use of systemic therapies in mCRPC patients who failed standard treatments, because: (3.3.1 - 3.3.4)		
3.3.1	Anti-tumor activity with PARPi, *e.g.* olaparib, rucaparib, niraparib, talazoparib etc., was seen in mCRPC patients with HRR mutations.	(5, 60–63)
3.3.2	Among various HRR mutations, tumors harboring *BRCA1/2m* appeared to derive the greatest clinical benefit from PARPi.	(5, 60, 61)
3.3.3	mCRPC patients with HRR mutations, in particular *BRCA2m*, who had prior androgen receptor-targeted agents ± chemotherapy should consider for olaparib (based on PROfound study) or other PARPi.	(5)
3.3.4	Patients with MSI-high or MMR-deficient tumors may have potential clinical benefit with immune-checkpoint inhibitors, *e.g.* pembrolizumab.	(64–66)
3.3.5	To derive optimal treatment benefits, all mCRPC patients should be tested for actionable genetic mutations.	(19)
3.3.6	Somatic testing is the preferred method for testing actionable genetic mutations.	–
3.3.7	Platinum-based chemotherapy may have anti-tumor activity in mCRPC patients with HRR mutations (*e.g. BRCA1/2m*).	(67, 68)

BRCAm, BRCA gene mutation; FH, family history; HRR, homologous recombination repair; mCRPC/mHSPC, metastatic castration-resistant/hormone-sensitive prostate cancer; MMR, mismatch repair; MSI, microsatellite instability; NGS, next-generation sequencing; PARPi, poly adenosine diphosphate-ribose polymerase inhibitors.

**Table 2:** Summary of panel consensus on genetic testing in prostate cancer (PC) according to disease status.

Disease Status	Panel Consensus	Statement(s)
Healthy germline *BRCA2m* carriers	Discuss cancer surveillance and prophylactic measures with patient; Start PC screening at age 40 years, or 10 years before the youngest PC diagnosis in family.	1.2.2 – 1.2.4
Any newly diagnosed PC	Obtain FH of prostate and related cancers.	1.1.1, 1.2.1
Localized PC	Some tissue-based genetic assays can provide useful information for detailed risk assessment.	3.1.2
with *BRCA2m*	- Active surveillance should not be offered.	3.1.1
Any PC with ductal or intraductal histology	Consider germline testing upon diagnosis, coupled with informed consent and genetic counseling.	1.3.1
mHSPC	Consider germline testing upon diagnosis, coupled with informed consent and genetic counseling; Genetic testing may help prognostic counseling and longer-term treatment planning.	1.3.1, 3.2.1
mCRPC	Perform somatic testing for detecting actionable mutations; - Potentially inherited mutations should be subject to germline confirmation with blood sample.	2.1.1, 2.2.1, 2.2.2, 3.3.5, 3.3.6
with FH	- Consider germline testing, coupled with informed consent and genetic counseling;	1.1.1, 1.3.1, 1.4.1, 2.1.2
with HRR mutation	- Platinum-based chemotherapy may have anti-tumor activity. - Patients who had prior androgen receptor-targeted agent(s) ± chemotherapy should consider olaparib or other PARPi.	3.3.3, 3.3.7
MSI-high or MMR-deficient	- May benefit from immune-checkpoint inhibitors, e.g. pembrolizumab.	3.3.4

BRCAm, BRCA gene mutation; FH, family history; HRR, homologous recombination repair; mCRPC/mHSPC, metastatic castration-resistant/hormone-sensitive prostate cancer; MMR, mismatch repair; MSI, microsatellite instability; PARPi, poly adenosine diphosphate-ribose polymerase inhibitors.

### 3.1 Part 1: Indications for Genetic Testing in PC

#### 3.1.1 FH of PC and Related Cancers

The FH criteria (Statement 1.1.1 in **Table 1**) were modified from the National Comprehensive Cancer Network (NCCN)’s Prostate Cancer Early Detection guidelines (17) and Philadelphia Prostate Cancer Consensus 2019 (18), with considerations of the local age of PC onset (2). The exclusion of Grade Group 1 (*i.e.* low grade) PC followed that in the NCCN guidelines. Also, in a multicenter sequencing analysis of 82 mPC patients with pathogenic DNA repair gene mutations, only two had a Gleason score of ≤ 6 (3).

#### 3.1.2 Familial Risks of PC

Taking FH is a definitive part of the clinical investigation of PC patients (1.2.1). In a U.S. survey of 132 urologists (21), 98% asked about FH of PC, but only some inquired about other malignancies associated with PC; *e.g.*, 76%, 52% and 48% asked about breast, ovarian and colorectal cancers, respectively. Also, 78% and 62% of respondents were aware of the association between hereditary PC and *BRCA2* mutations (*BRCA2m*) and *BRCA1* mutations, respectively, but < 40% were aware of the association between hereditary PC and Lynch syndrome.

Through the increasing availability of germline testing, mutation carriers can be identified to facilitate early PC detection (1.2.2). Alternatively, patients with a personal history of related cancers may be referred for genetic testing and counselling through the relevant guideline recommendations, such as for male breast cancer (69). For germline mutation carriers, the associated personal and familial implications should be explained clearly, and should include associated cancer risks and ways to cope with potential stress and anxiety (see also *Consent and genetic counseling*).

For *BRCA2m* carriers (including those with PC), the NCCN Genetic/Familial High-Risk Assessment guidelines (70) recommend clinical breast examination starting at 35 years of age, pancreatic cancer screening (for those with FH of pancreatic cancer), and general melanoma risk management. For those with *MLH1* mutations, the NCCN guidelines (71) recommend pancreatic cancer screening (for those with FH of pancreatic cancer), high-quality colonoscopy (every 1–2 years), and baseline esophagogastroduodenoscopy with random biopsy (every 3–5 years).

Although the usual age of PC onset may be later in Asian compared with Western populations (72), it is unclear whether this difference applies to mutation carriers. For example, the *HOXB13* G84E mutation has been associated with early PC onset in Caucasian populations. Although the mutation is rarely detected in Asian men, alternative similar mutations have been reported, including G135E and G132E in Chinese and Japanese studies, respectively (73). Thus, further studies are needed to elucidate the prevalence and effects of these recurrent mutations on PC onset. In the 3-year interim report of IMPACT (22), a longitudinal study of 1,821 *BRCA1/2* mutation carriers and 1,206 noncarriers, the median age of PC diagnosis was 61 years in the *BRCA2m* carrier arm and 64 years in the noncarrier arm (*p* = 0.04). In Hong Kong, most PC cases occur in patients over age 50 (2). Because of the increased aggressiveness of PC in *BRCA2m* carriers, early screening is recommended (1.2.3, 1.2.4).

#### 3.1.3 Germline Testing Upon PC Diagnosis

The panel recommends germline testing in PC patients with metastatic disease (1.3.1), ductal or intraductal histology (1.3.2), or positive FH (1.3.3), because significantly increased rates of germline mutations have been reported in these populations. Studies suggest that germline mutation prevalence is much higher in men with mPC (11.8%) than those with localized PC (4.6%), or compared with healthy individuals (2–3%) (74). Also, PC patients with germline mutations were more likely to harbor intraductal/ductal histology than those without (48% vs. 12%, *p* < 0.01) (24), and ~50% of patients with prostatic ductal adenocarcinoma had DNA repair gene alterations (25).

#### 3.1.4 Consent and Genetic Counseling

With regard to the manifold implications of germline testing, it is good clinical practice to require a detailed informed consent, in addition to the written consent required by the testing laboratory (1.4.1). Familial risk assessment should include a Bayesian analysis performed by a qualified genetic counselor. For germline testing indicated after somatic testing (19), prior consent may not be sufficient. The panel recommends discussing the following items during the consent process: testing purpose, panel choices, cost, potential implications on disease management, additional risks, health insurance, legal issues, and potential significance to family members (18).

Because of limitations in resources and training, urologists and oncologists may not be in the best position to provide specialized genetic counseling to patients. In Hong Kong, there exists a pressing need for professional genetic counselors in this area (1.4.2).

#### 3.1.5 Hereditary Driver Mutations

In clinical practice, the number of genes selected for germline testing will depend on the choice of testing method and associated cost. For Asian patients, we suggest including the genes mentioned in Statement 1.5.1 in a multi-gene panel. Mutations in these genes possess potential prognostic and therapeutic significance (18, 19), and have been reported in studies involving Asian patients (33, 73). For example, mutations in the DNA mismatch repair (MMR) genes *MLH1, MSH2, MSH6* and *PMS2* have been reported in Chinese patients with Lynch syndrome, at 2–14% prevalence (75). We specifically did not include *CHEK2* and *HOXB13* because they appear to be rare in Chinese populations (76, 77) and, to our knowledge, no large study has reported on *CDK12* germline mutations in Asian PC patients (see also *Testing levels and coverage*).

#### 3.1.6 Ethnic Considerations

In Hong Kong, the Hong Kong Hereditary Breast Cancer Family Registry helps to archive the incidence of hereditary mutations for high-risk breast and ovarian cancers, including *BRCA1/2* mutations (28). A similar registry is needed for hereditary PC (1.6.1). While variations exist between different regions of Asia (73), local studies in Hong Kong would be valuable, particularly the detection and classification of uncertain variants (34).

### 3.2 Part 2: Testing Methods and Technical Considerations

#### 3.2.1 Germline Versus Somatic Testing

A landmark study on the genomics of advanced PC (4) showed that, among 150 mCRPC patients, 19/150 (12.7%) had *BRCA2* loss, of which ~90% was biallelic. Eight (5.3%) affected individuals harbored pathogenic germline *BRCA2m*, with a later somatic event leading to biallelic loss. A subsequent study by the New York Memorial Sloan Kettering Cancer Center (78) demonstrated that, in 504 tumors from 451 PC patients, of whom 221 consented to germline testing, the incidence of any alteration in *BRCA1/2*, *ATM* and *CHEK2* was 27%. These included germline mutations (*BRCA2*, 8.6%; *BRCA1*, 0.9%; *ATM*, 2.3%; *CHEK2*, 4.1%) and somatic mutations only (*BRCA2*, 7.7%; *BRCA1*, 0.9%; *ATM*, 4.5%; *CHEK2*, 0.9%). These studies suggest that germline mutations involving DNA repair genes account for almost half of all detected mutations through somatic testing of PC tissue.

Because both germline and somatic mutations are relevant to the molecular pathology of PC, genetic testing can proceed in two orders: 1) germline testing, after genetic counseling and informed consent (*e.g.* patients with FH); or 2) somatic testing of tumor tissue, after explaining to patients that any detected potential germline mutation may require further confirmatory testing (38). When an HRR or MMR mutation is detected, it will be important to undergo germline confirmation by testing a peripheral blood sample, so that proper genetic counseling and cascade testing can be offered to family members (2.1.1).

In a Hong Kong ovarian cancer consensus (28), opinion favored performing somatic testing before germline testing. In this order, the patient can decide whether to undergo testing solely for the purpose of informing treatment planning, without having to understand the potential hereditary implications in advance. Germline testing can be considered after treatment decision has been made. However, one disadvantage of this approach is that cascade testing of family members is more easily missed or forgotten (28).

A large-scale cross-sectional study (79) investigated the data of 3,607 men with a personal history of PC who underwent germline testing between 2013 and 2018. After examining the self-reported FH data, 37% of patients with positive detectable variants would not have been approved for germline testing using NCCN guidelines criteria. This suggests that DNA repair gene variants detected by somatic testing in PC should be considered for germline confirmation, irrespective of FH or Gleason score.

##### 3.2.1.1 Large Genomic Rearrangements (LGRs)

LGRs are large, usually exon-level deletions, or other structural variations that span megabase segments of the human genome (39). A Hong Kong study (80) of 1,236 patients with high-risk hereditary breast and/or ovarian cancers showed that, among 120 deleterious *BRCA* mutations, 8 (6.7%) were LGRs involving *BRCA1* (5/57, or 8.8%) and *BRCA2* (3/63, or 4.8%). LGRs are not detectable by Sanger sequencing, and require multiple ligation-dependent probe amplification (MLPA) for detection (81). With the advancement of multi-gene next-generation sequencing (NGS) panels for germline testing, using molecular barcodes and specialized bioinformatics methods, large deletions have become detectable by coverage depth analysis, thus obviating routine MLPA.

Detection of LGRs in somatic testing using NGS on formalin-fixed paraffin-embedded (FFPE) tissue is challenging. Indeed, not all NGS methods are developed or optimized for the detection of copy number variants. For example, an inter-laboratory study (82) of FFPE tumor DNA samples showed that the large insertion *BRCA1* exon13ins6kb, a known pathogenic variant, was not detected by any of the laboratories in the primary analysis. If a patient has FH, or is otherwise suspected of having inherited cancer, germline testing using a peripheral blood sample should be considered, even if NGS returned negative results (2.1.2). Various options of platforms and assays for PC genetic testing are available from different testing laboratories. Strengths and limitations of the available panels were discussed in Giri *et al.* (18).

#### 3.2.2 Tissue Sample Availability

In the PROfound study, multi-gene NGS assays successfully sequenced and produced biomarker status outcomes in only 69% of tumor samples, and the success rate dropped to about 50% in tissue specimens that were stored for 5 or more years (41). This was comparable to those in recent trials involving somatic testing of tumor tissues. Major reasons for testing failure include a limited amount of tumor tissue collected during diagnostic biopsy, exhaustion of diagnostic material during histological examination, insufficient tumor content for genomic study, and suboptimal DNA quality and/or quantity due to DNA degradation during tissue fixation and storage (83).

The histopathologist is in a key position to optimize the success rate of multi-gene NGS assay in PC (2.2.1). FFPE samples for HRR gene mutation testing should contain sufficient cellularity (> 5,000 cells is equivalent to ~30 ng of DNA) to yield the required amount of DNA for testing (84). A minimum of neoplastic cell content is also required; *i.e.* tumor content should be at least 10–20% to enable reliable detection of somatic variants at > 5% allele frequency, and higher for detection of LGRs. Other recommendations on processing and storage of FFPE samples for DNA analysis in PC can be found in Gonzalez *et al.* (84).

#### 3.2.3 Testing Levels and Coverage

Genomic profiling of PC is performed by multi-gene NGS panels that comprehensively cover all types of genetic variants; namely, single nucleotide variants, small insertion/deletions, copy number variants including large deletions, and gene fusions (2.3.1). To the clinician, the most important variants are actionable mutations that can guide treatment decisions. A real-world study (85) on clinical comprehensive genomic profiling in 3,476 clinically advanced PCs (1,660 primary tumors and 1,816 metastases) showed that potentially targetable genomic alterations were frequently identified in DNA damage repair, phosphatidylinositol-3-kinase (PI3K), and RAS/RAF/MEK pathways, among other genetic changes. Among the DNA damage repair pathways, homologous recombination defects (HRDs) were found in 23.4% of cases, Fanconi anemia gene defect in 4.8%, *CDK12* abnormality in 5.6%, and MMR gene defects in 4.3%.

##### 3.2.3.1 Ethnic Variations

Genetic mutations vary with ethnicity. The spectrum of mutations seen in Chinese PC patients differs from that observed in Caucasian populations. As the majority of participants in large-scale international studies were Western populations, some commonly reported mutations may, in fact, be rare in Chinese PC patients. For example, the most frequently reported mutation of the *CHEK2* gene, c.1100delC (3), has not been found in Chinese studies. Likewise, while the *HOXB13* gene is believed to be an uncommon cause of familial PC in European populations (86), the *HOXB13* G84E hotspot missense mutation was not detected in a study of 1,123 patients from 18 centers across China (76). A study of breast and ovarian cancer patients from Southern China reported a unique set of recurrent or founder mutations of *BRCA* (87). Thus, annotation of genetic variants detected by multi-gene NGS panels should consider ethnic origins.

##### 3.2.3.2 HRR Gene Mutations

The presence of HRR gene mutations in a mCRPC patient helps to predict response to PARPi treatment (6). Also, MMR gene mutations, which are associated with the microsatellite instability (MSI)-high (MSI-H) phenotype, increase susceptibility to immune checkpoint inhibitor (ICI) treatment, such as programmed death-1 (PD-1) blockade (88). For PC patients, DNA repair genes of interest include: HRR genes (most significant: *BRAC2*, *BRCA1*, *ATM*; others: *ATR*, *BARD1*, *BRIP1*, *CHEK1*, *CHEK2*, *FAM175A*, *GEN1*, *MRE11A*, *PALB2*, *PPP2R2A*, *NBN*, *RAD31*, *RAD51B*, *RAD51C*, *RAD51D* and *RAD54L*); MMR genes (*MSH2*, *MSH6*); Fanconi anemia genes (*FANCA*, *FANCL*); and *CDK12* (3–5, 60). For mPC, the Philadelphia 2019 consensus (18) recommends large panels and somatic testing; the genes considered were *BRCA1*/*2*, *HOXB13*, *CHEK2*, *ATM*, *NBN*, *MSH2*, *MSH6*, *MLH1*, *PMS2*, *BRIP1*, *TP53* and the Fanconi anemia genes.

For determining eligibility for PARPi therapy, it should be noted that gene-level testing alone, whether germline or somatic, cannot cover all HRDs (45) (2.3.2). Hence, in addition to HRR gene mutation testing, HRD testing by genomic signatures and scars, such as the genomic instability score or loss of heterozygosity (LOH) score from commercial laboratories may need to be considered, to better stratify patients and inform treatment selection (89). Efforts to standardize and harmonize these and other similar assays are needed. Novel techniques, including mutational signatures and functional assays for HRD testing, are currently under research (46).

##### 3.2.3.3 MMR Gene Mutations

DNA MMR is a system to correct the erroneous insertion, deletion and mismatch of bases that can occur during DNA replication and recombination. Because the accumulation of mismatches leads to DNA disruption and cell death, MMR is essential for maintaining genomic stability. Major MMR genes in humans include *MLH1*, *MSH2*, *MSH6* and *PMS2*.

A large amount of MMR defects can be characterized by a “MSI-H” phenotype. Conventionally, polymerase chain reaction (PCR) and capillary electrophoresis of two single-nucleotide repeat loci, and three multi-nucleotide repeat loci on paired normal tumor samples, are employed to determine MSI status (90) (2.3.3). Instability of one locus is termed “MSI-low (MSI-L),” instability of ≥ 2 loci is “MSI-H,” and “microsatellite-stable (MSS)” denotes that no loci have been affected.

Currently, MSI status is detected by NGS cancer genomic profiling, which is applicable to PC (47). Apart from MMR gene mutations, epigenetic changes such as *MLH1* gene promoter hypermethylation can also cause sporadic or acquired MSI. Using a routine immunohistochemistry (IHC) panel of antibodies against MLH1, MSH2, MSH6 and PMS2, defective MMR status in PC may also be determined indirectly by the loss of MMR protein expression on tumor cells (91). The use of IHC is a reliable and cost-effective way of determining MMR status.

MMR defects are identified in ~5% of mPC, of which the most frequently mutated genes are *MSH2* and *MSH6*; the majority (~75%) of these mutations occur at the somatic level and are not inherited (92). Nevertheless, as mentioned in Statement 2.1.1, MMR gene mutations detected by an NGS tumor panel should be subject to germline confirmation by testing a peripheral blood sample.

##### 3.2.3.4 ICI Effect Biomarkers

There is growing evidence that tumor mutational burden (TMB), MSI status and programmed death ligand-1 (PD-L1) expression are biomarkers that can help to predict response to ICIs (2.3.4). In 2017, the US Food and Drug Administration (FDA) granted accelerated approval of the ICI pembrolizumab for treating patients with unresectable or metastatic MSI-H or MMR-deficient (dMMR) solid tumors who progressed on prior therapy with no satisfactory treatment alternative (88). This was also the FDA’s first tissue/site-agnostic approval for anti-cancer therapy. In 2020, a similar accelerated approval was granted for use of pembrolizumab in patients with advanced solid tumors that display high TMB, defined as ≥ 10 mutations/megabase in an FDA-approved test (93).

One caveat is that these biomarkers do not correlate well with one another. In a study of 11,348 cases across 26 types of cancer (49), 3.0%, 7.7% and 25.4% were MSI-H, TMB-high and PD-L1-positive, respectively. Of note, 30% of MSI-H cases were TMB-low, suggesting that the two biomarkers are not diagnostically interchangeable. This result might have been expected, because MSI focuses on specific genomic regions known to accumulate errors, whereas TMB covers the genome more broadly. Hence, MSI-H tumors are likely to be TMB-high, but not *vice versa.* In addition to genomic biomarkers (*e.g.* MSI and TMB), inflammatory biomarkers such as PD-L1 and T cell–inflamed gene expression profile (GEP) may also help to stratify patients by predicted clinical response (94). Because MSI prevalence in PC is low (~3%), and data for the reliability of IHC and PCR outside of Lynch syndrome are lacking, the European Society for Medical Oncology guideline recommends using NGS for MSI testing in PC patients (50).

##### 3.2.3.5 Liquid Biopsy

In patients with advanced or mPC, especially in the castration-resistant state, it may be difficult to obtain tissue biopsy samples with a sufficient yield; for example, in those with bone-predominant metastases. Alternatively, using archival tissues from the primary prostatic biopsy presents significant limitations because such tissues may fail to reflect the latest tumor mutations, and the DNA yield and quality will be reduced, depending on storage duration and condition (see *Tissue sample availability*). Liquid biopsy using ctDNA is an emerging alternative method to capture tumor features (2.3.5). This DNA is shed into the bloodstream by degrading tumor cells, and its yield increases with disease burden and active progression (95). In a sequencing study of 72 genes from 45 ctDNA samples from mPC patients (9), prostate-specific antigen (PSA) concentrations did not differ in patients with or without detectable ctDNA, although patients with high PSA levels (> 370 ng/mL) all had detectable ctDNA. Consistent with previous studies, high ctDNA fractions were associated with poor prognosis and correlated with overall tumor burden (9).

In the PROfound study, *BRCA* and *ATM* mutations identified in ctDNA showed 81% positive percentage agreement and 92% negative percentage agreement with matched tumor tissues (8). Moreover, liquid biopsy may capture spatial tumor heterogeneity and temporal tumor evolution (96). Unlike a single tissue biopsy, liquid biopsy can integrate genomic alterations from more than one metastatic lesion and thereby survey any tumor heterogeneity in the patient, which may help to detect pathway alterations and resistance mechanisms (96).

### 3.3 Part 3: Indications for Genetic Testing in PC

#### 3.3.1. Localized PC Patients

AS is a management option offered to patients with low-risk localized PC to defer curative-intent treatment in order to reduce over-treatment and treatment-related complications. This is usually applied to patients with International Society of Urological Pathology (ISUP) Grade Group 1, clinical stage T1c or T2a, PSA level < 10 ng/mL and PSA density < 0.15 ng/mL/cc (97). The presence of a germline *BRCA2m* is an independent, poor prognostic factor for localized PC, with increased risks of an aggressive phenotype and metastasis (98), shortened survival (99), and grade reclassification during AS (7). The Philadelphia consensus (18) encourages germline *BRCA2* testing for AS discussions. In our opinion, current evidence does not warrant routine germline testing in all localized PC patients. However, we agree that patients with otherwise known germline mutations should not be offered AS, due to the higher risks of disease progression as mentioned above (3.1.1).

In localized PC patients, risk stratification usually includes consideration of PSA level, Gleason score, and clinical T-staging, each of which may have their own limitations. Genetic analysis can provide additional information on the molecular basis of the condition (3.1.2). Notable early events may include inactivation of tumor suppressors, cell cycle dysregulation, and rearrangement between androgen responsive genes (*e.g. TMPRSS2*) and the ETS transcription factor family of genes (most commonly *ERG*) (100). The Prolaris assay reports a cell cycle progression score that predicts the risk of cancer-specific mortality (52), biochemical recurrence after external beam radiotherapy (53), and outcomes after radical prostatectomy (54). The Decipher test predicts metastatic progression (55), including post-radical prostatectomy metastasis (56), cancer-specific mortality (101), and post-operative radiation sensitivity (102). Note that due to the low reported rates of genetic mutations in localized PC patients (see *Germline testing upon PC diagnosis*), our panel did not reach a consensus on genetic testing for driver mutations in this group (**Supplementary Table 5** and **Appendix B**).

#### 3.3.2 Genetic Testing in mHSPC

In addition to androgen deprivation therapy, there is growing evidence to support the use of chemotherapy (docetaxel) or an androgen receptor-signaling inhibitor to provide further survival benefit in mHSPC patients (103–105). In mHSPC patients receiving standard therapy, preliminary studies suggest that the presence of DNA repair gene mutations has not been associated with OS, progression-free survival (PFS) or response rate (58, 106), but was associated with early progression to CRPC (59). For patients with *de novo* mHSPC, genetic testing may provide additional information for prognostic counseling and longer-term treatment planning (3.2.1).

#### 3.3.3 Systemic Therapies for mCRPC

We suggest that all mCRPC patients undergo genetic testing for actionable mutations (3.3.5), which may help to identify eligibility for systemic treatments, including PARPi (3.3.1–3.3.3), ICIs (3.3.4), and platinum-based chemotherapy (3.3.7). Due to potential resource limitations and psychological burden on the patient, somatic testing may initially be preferred (3.3.6; see also *Germline versus somatic testing*). It may be worth noting that eligibility to PARPi for PC patients, in terms of clinical trial inclusion or the approved indications (6, 107), has so far included both germline and somatic mutations.

##### 3.3.3.1 PARPi

PARP is an enzyme that facilitates DNA repair by binding to the site of DNA damage and attracting DNA repair proteins (108). Inhibition of PARP leads to accumulation of DNA damage, genomic instability, and ultimately cell death – a mechanism that has been termed “synthetic lethality” (109). HRR mutations result in the utilization of more error-prone pathways, and thereby increase susceptibility to PARP inhibition.

PROfound (5) was a phase 3 open-label randomized trial comparing the PARPi olaparib with physician’s choice of a new hormonal agent (NHA; abiraterone or enzalutamide) in 387 mCRPC patients with ≥ 1 HRR mutation (Cohort A: *BRCA1/2* or *ATM* mutations; Cohort B: 12 other prespecified genes), who progressed while receiving an NHA. In Cohort A, the primary endpoint of median radiographic PFS (rPFS) was significantly improved in the olaparib arm versus controls (7.4 vs. 3.6 months; hazard ratio [HR] = 0.34, *p* < 0.001); objective response rate (ORR) and time to pain progression were also significantly greater in the olaparib arm. In the overall population (Cohorts A and B), median rPFS was also significantly improved in the olaparib arm versus controls (5.8 vs. 3.5 months; HR = 0.49, *p* < 0.001). Encouragingly, the final median OS (61) also demonstrated a significant benefit in Cohort A versus the NHA arm (19.1 vs. 14.7 months; HR = 0.69, *p* < 0.02).

TRITON2 (60) was a phase 2 open-label trial of rucaparib in 115 mCRPC patients with *BRCA* mutation who progressed after one or two lines of androgen receptor-directed therapy and one taxane-based chemotherapy. The ORR (primary endpoint) was 43.5%, with median rPFS of ~9 months and confirmed PSA response rate of 54.8%.

##### 3.3.3.2 ICIs

PC is recognized as poorly immunogenic, showing low levels of T-cell activation and high levels of immunosuppressive activities (110). The 2017 FDA approval of pembrolizumab for treating unresectable or metastatic MSI-H or dMMR solid tumors (88) was based on the KEYNOTE-158 results, which included 2 mCRPC patients, of whom one had a partial response and the other had stable disease for > 9 months (111). In the open-label phase 2 KEYNOTE-199 study of pembrolizumab in 258 patients with mCRPC regardless of MSI status (64), 9% achieved ≥ 50% PSA response, and 3–4% had an objective radiographic response. In a retrospective study of 1,033 mCRPC patients (65), of 11 MSI-H/dMMR patients who received ICI therapy, 6 (55%) had ≥ 50% PSA response and 4 showed radiographic response.

##### 3.3.3.3 Platinum-Based Chemotherapy

Studies in breast and ovarian cancers have shown that patients with *BRCA1/*2 mutations are responsive to platinum-based chemotherapy (112). For PC patients, Cheng *et al.* (113) reported exceptional response to platinum-based chemotherapy in 3 patients with biallelic *BRCA2* inactivation, despite disease progression on prior standard therapies. In a retrospective analysis of 141 mCRPC patients who received ≥ 2 doses of carboplatin and docetaxel (112), 6 of 8 (75%) of *BRCA2m* carriers experienced PSA declines > 50% within 12 weeks, compared with 17% of noncarriers (*p* < 0.001). In a multicenter retrospective analysis of 508 CRPC patients treated with platinum-based chemotherapy (67), those with DNA repair gene aberrations showed higher rates of PSA declines and soft tissue responses (vs. those without), although the differences were not statistically significance (*p* = 0.20 and *p* = 0.07, respectively). Among those with *BRCA2m*, large proportions demonstrated PSA declines of ≥ 50% or soft tissue responses (63.9% and 50% of 44 patients, respectively). Although the evidence is derived from retrospective studies, it has consistently suggested that platinum-based chemotherapy may have anti-tumor activity in mCRPC patients with HRR mutations (3.3.7).

## 4 Discussion

### 4.1 Further Practical Advice

Among the currently available testing methods, somatic testing using tumor tissue samples has the highest yield in detection of actionable genetic mutations, and may lead to new treatment options for the patient. However, it is important for clinicians to know the appropriate scope to test for, including HRR gene mutations (e.g. *BRCA1/2, ATM*, etc.), MMR gene mutations (e.g. *MLH1, MSH2, MSH6, PMS2)*, presence of MSI, and TMB. Clinicians should note the following important technical aspects in genetic testing: 1) tumor testing success rates are reduced with longer storage times; 2) performing only somatic (tumor) testing may not necessarily reflect germline mutation status; 3) ctDNA is a convenient alternative for tumor testing; and 4) if a germline mutation was detected, proper genetic counseling provided by trained personnel is ideal, to inform the patient’s family members about hereditary risks. Cascade testing in PC can be challenging, as PC is not traditionally considered an inherited cancer, and is not usually an “index” cancer diagnosed in families with Lynch syndrome (114). Men may also be less likely than women to undergo testing (115).

### 4.2 Limitations

With emerging evidence and recommendations from international guidelines on the indications and scope of genetic testing for PC, this consensus aims to provide a local guide to urologists, oncologists and pathologists on who and what to test for in selected patients. In areas with limited data in Asian men, evidence from Caucasian studies and international guidelines were used for reference. Also, the term “Asian” is relatively broad, and the data mentioned here should not be taken as absolute values, but as reminders to clinicians of the ethnic and region-specific factors involved in treating Asian patients. More large-scale sequencing studies are warranted to identify ethnic-specific variants and better understand their pathophysiological effects.

## 5 Conclusion

This consensus provides guidance to urologists, oncologists and pathologists working with Asian patients on the indications for genetic testing, testing methods and technical considerations, and associated therapeutic implications.

## Data Availability Statement

The original contributions presented in the study are included in the article/**Supplementary Material**. Further inquiries can be directed to the corresponding author.

## Author Contributions

The panel meetings were co-chaired by DP and PC. All authors participated in the panel meetings, contributed to drafting the consensus statements and journal manuscript, and read and approved the final version.

## Funding

This study did not receive any specific grants from funding agencies in the public, commercial, or not-for-profit sectors. Editorial assistance was provided by Best Solution, which was funded by the Hong Kong Society of Uro-Oncology.

## Conflict of Interest

The authors declare that the research was conducted in the absence of any commercial or financial relationships that could be construed as a potential conflict of interest.

## Publisher’s Note

All claims expressed in this article are solely those of the authors and do not necessarily represent those of their affiliated organizations, or those of the publisher, the editors and the reviewers. Any product that may be evaluated in this article, or claim that may be made by its manufacturer, is not guaranteed or endorsed by the publisher.
