# Supplementary material for: Genetic Testing and Its Clinical Application in Prostate Cancer Management: Consensus Statements from the Hong Kong Urological Association and Hong Kong Society of Uro-Oncology
Source: Front Oncol. 2022 Jul 18;12:962958. doi: 10.3389/fonc.2022.962958 (PMC9339641; doi:10.3389/fonc.2022.962958)
Supplement: Supplementary file 1 [file DataSheet_1.docx]

**Supplementary Table 1.** Panelists and their affiliations.

| Panelist | Specialty | Qualifications | Association | Affiliations |
| --- | --- | --- | --- | --- |
| Dr. Darren M. C. POON (Co-chairperson) | Oncology | MBChB (CUHK), FRCR, FHKCR, FHKAM (Radiology) | HKSUO | Honorary Clinical Associate Professor, Department of Clinical Oncology, The Chinese University of Hong Kong |
| Prof. Peter K. F. CHIU (Co-chairperson) | Urology | MBChB (CUHK), PhD (Eur), FRCSEd (Urol), FCSHK, FHKAM (Surg) | HKUA | Associate Professor, Department of Surgery, The Chinese University of Hong Kong |
| Dr. Marco T. Y. CHAN | Urology | MBBS (HK), FRCSEd (Urol), FCSHK, FHKAM (Surg) | HKUA | Associate Consultant, Division of Urology, Department of Surgery, Tuen Mun Hospital, Hong Kong |
| Dr. Wilson H. C. CHAN | Urology | MBChB (CUHK), FRCSEd (Urol), FCSHK, FHKAM (Surg) | HKUA | Associate Consultant, Department of Surgery, United Christian Hospital, Hong Kong |
| Dr. M. H. CHEUNG | Urology | MBChB (CUHK), FRCSEd (Urol), FCSHK, FHKAM (Surg) | HKUA | Associate Consultant, Division of Urology, Department of Surgery, Tseung Kwan O Hospital, Hong Kong |
| Dr. Martin H. C. LAM | Oncology | MBBS (HK), FRCR, FHKCR, FHKAM (Radiology) | HKSUO | Associate Consultant, Department of Oncology, United Christian Hospital, Hong Kong |
| Dr. Eric K. C. LEE | Oncology | MBChB (CUHK), FRCR, FHKCR, FHKAM (Radiology) | HKSUO | Department of Clinical Oncology, Tuen Mun Hospital, Hong Kong |
| Dr. Edmond S. K. MA | Pathology | MBBS (HK), MD (HK), MRCP (UK), FRCP (Edin), FRCP (Lond), FRCP RCPS (Glasg), FRCPath, FRCPA, FHKCPath, FHKAM (Pathology) | Guest panelist | Honorary Clinical Associate Professor, Department of Pathology, The University of Hong Kong  Director, Clinical Pathology & Molecular Pathology Division, Hong Kong Sanatorium & Hospital |

Abbreviations: HKSUO, Hong Kong Society of Uro-Oncology; HKUA, Hong Kong Urological Association.

**Supplementary Table 2.** Meeting dates and brief agendas.

| Meeting | Format | Date | Agenda |
| --- | --- | --- | --- |
| 1 | Virtual | December 16, 2020 | - Defining the scope of study - Formalizing the study method - Literature overview - Dividing panel subgroups |
| 2 | Virtual | March 29, 2021 | - Presentations: Parts 1 and 3 - Discussions: Parts 1 and 3 |
| 3 | Physical + Virtual | May 5, 2021 | - Presentations: Part 2 - Discussions: Part 2 |
| 4 | Physical | June 2, 2021 | - Further discussions: Parts 1 to 3 |
| 5 | Virtual | June 25, 2021 | - Voting and review of results |

**Supplementary Table 3.** Level of evidence and grade of recommendation criteria; modified from (1).

| Level of Evidence | | Grade of Recommendation | | Practicability of Recommendation | |
| --- | --- | --- | --- | --- | --- |
| I | Evidence obtained from at least 1 randomized controlled trial | A | There is good evidence to support the statement | A | Accept completely |
| II-1 | Evidence obtained from well-designed control trials without randomization | B | There is fair evidence to support the statement | B | Accept with some reservation |
| II-2 | Evidence obtained from well-designed cohort or case-control study | C | There is poor evidence to support the statement, but recommendation made on other ground(s) | C | Accept with major reservation |
| II-3 | Evidence obtained from comparison between time or places, with or without intervention | D | There is fair evidence to refute the statement | D | Reject with reservation |
| III | Opinion of respected authorities, based on clinical experience and expert committee | E | There is good evidence to refute the statement | E | Reject completely |

**Supplementary Table 4.** Statement voting results: a) individuals; b) totals.

a)

| Candidate Statements | Responses (%) | | | | | Accept (A + B ≥ 75%) or  Reject (A + B < 75%) | |
| --- | --- | --- | --- | --- | --- | --- | --- |
|  | A | B | C | D | E | A + B % | Outcome |
| Part 1: Indications for Genetic Testing in PC | | | | | | | |
| 1.1 FH of PC and Related Cancers | | | | | | | |
| 1.1.1 | 100% | 0% | 0% | 0% | 0% | 100% | Accept |
| 1.2 Familial Risks of PC | | | | | | | |
| 1.2.1 | 100% | 0% | 0% | 0% | 0% | 100% | Accept |
| 1.2.2 | 100% | 0% | 0% | 0% | 0% | 100% | Accept |
| 1.2.3 | 25% | 63% | 13% | 0% | 0% | 88% | Accept |
| 1.2.4 | 50% | 38% | 13% | 0% | 0% | 88% | Accept |
| 1.2.5 | 13% | 50% | 38% | 0% | 0% | 63% | Reject |
| 1.3 Germline Testing Upon PC Diagnosis | | | | | | | |
| 1.3.1* | 75% | 13% | 13% | 0% | 0% | 88% | Accept |
| 1.3.2* | 38% | 38% | 13% | 13% | 0% | 75% | Accept |
| 1.3.3* | 63% | 38% | 0% | 0% | 0% | 100% | Accept |
| 1.3.4* | 13% | 0% | 13% | 50% | 25% | 13% | Reject |
| 1.3.5* | 25% | 13% | 25% | 38% | 0% | 38% | Reject |
| 1.3.6* | 38% | 13% | 25% | 25% | 0% | 50% | Reject |
| 1.4 Consent and Genetic Counseling | | | | | | | |
| 1.4.1 | 75% | 25% | 0% | 0% | 0% | 100% | Accept |
| 1.4.2 | 100% | 0% | 0% | 0% | 0% | 100% | Accept |
| 1.5 Hereditary Driver Mutations | | | | | | | |
| 1.5.1 | 100% | 0% | 0% | 0% | 0% | 100% | Accept |
| 1.6. Ethnic Considerations | | | | | | | |
| 1.6.1 | 100% | 0% | 0% | 0% | 0% | 100% | Accept |
| Part 2: Testing Methods and Technical Considerations | | | | | | | |
| 2.1 Germline vs. Somatic Testing | | | | | | | |
| 2.1.1 | 88% | 0% | 13% | 0% | 0% | 88% | Accept |
| 2.1.2 | 75% | 25% | 0% | 0% | 0% | 100% | Accept |
| 2.2 Tissue Sample Availability | | | | | | | |
| 2.2.1 | 100% | 0% | 0% | 0% | 0% | 100% | Accept |
| 2.2.2 | 100% | 0% | 0% | 0% | 0% | 100% | Accept |
| 2.3 Testing Levels and Coverage | | | | | | | |
| 2.3.1 | 50% | 50% | 0% | 0% | 0% | 100% | Accept |
| 2.3.2 | 63% | 38% | 0% | 0% | 0% | 100% | Accept |
| 2.3.3 | 100% | 0% | 0% | 0% | 0% | 100% | Accept |
| 2.3.4 | 100% | 0% | 0% | 0% | 0% | 100% | Accept |
| 2.3.5 | 100% | 0% | 0% | 0% | 0% | 100% | Accept |
| Part 3. Therapeutic Implications | | | | | | | |
| 3.1. Risk Assessment for Localized PC Patients | | | | | | | |
| 3.1.1 | 38% | 63% | 0% | 0% | 0% | 100% | Accept |
| 3.1.2 | 50% | 50% | 0% | 0% | 0% | 100% | Accept |
| 3.2 Genetic Testing in mHSPC | | | | | | | |
| 3.2.1 | 88% | 13% | 0% | 0% | 0% | 100% | Accept |
| 3.3 Systemic Therapies for mCRPC | | | | | | | |
| 3.3.1 | 88% | 13% | 0% | 0% | 0% | 100% | Accept |
| 3.3.2 | 100% | 0% | 0% | 0% | 0% | 100% | Accept |
| 3.3.3 | 100% | 0% | 0% | 0% | 0% | 100% | Accept |
| 3.3.4 | 88% | 13% | 0% | 0% | 0% | 100% | Accept |
| 3.3.5 | 88% | 13% | 0% | 0% | 0% | 100% | Accept |
| 3.3.6 | 88% | 13% | 0% | 0% | 0% | 100% | Accept |
| 3.3.7 | 75% | 25% | 0% | 0% | 0% | 100% | Accept |

*The statements in this section have been re-numbered according to acceptance/rejection for easier reading. Abbreviations: FH, family history; mCRPC / mHSPC, metastatic castration-resistant / hormone-sensitive prostate cancer; PC, prostate cancer.

b)

| Part | Accepted | Rejected |
| --- | --- | --- |
| 1 | 12 | 4 |
| 2 | 9 | 0 |
| 3 | 10 | 0 |
| Total | 31 | 4 |

**Supplementary Table 5.** Rejected statements on prostate cancer (PC) genetic testing.

| Rejected Statements | | Potential Ref.* |
| --- | --- | --- |
| 1.2 Familial Risks of PC | | |
| 1.2.5 | *HOXB13*, *BRCA1*, *ATM* and DNA MMR mutation carriers may consider a similar approach to above (R1.2.1–R1.2.4) | (2) |
| 1.3 Germline Testing Upon PC Diagnosis | | |
| Germline testing should be considered in PC patients with: | | |
| 1.3.4 | All PC patients. | (2) |
| 1.3.5 | Regional disease (cN1). | (3) |
| 1.3.6 | High-risk localized PC. | (4, 5) |

*The references cited here contain relevant information, but do not necessarily support the statement.

Abbreviations: MMR, mismatch repair.

**Appendix A.** HKUA and HKSUO background information.

Since its commencement in 1988, the Hong Kong Urological Association (HKUA) has been operating with two objectives: 1) facilitate academic discussions for the advancement of urology; and 2) equip the general public with a better understanding of urological diseases. Through our education efforts, we hope that patients with urological conditions will benefit from early intervention, and that disease prevention may be achieved by means of health promotion.

Founded in 2015, the Hong Kong Society of Uro-Oncology (HKSUO) aims to provide a platform for multi-specialty discussions and collaboration on urological cancers. In the past 6 years, the Society has played an important role locally in promoting clinicians’ understanding of the latest developments in the treatment and management of urological cancers, and in facilitating the application of cutting-edge treatment advancements.

**Appendix B.** Explanations for the rejected statements.

1.2.5: Of 20 DNA-repair genes assessed by Pritchard *et al.* (4) in 692 men with metastatic PC, *ATM* mutations were the second most common pathogenic germline mutations (11/84; 13%). In a retrospective study (6) of 1,123 Chinese PC patients from 18 centers across China, *ATM*, *BRCA1* and *HOXB13* pathogenic mutations were found in 1.3%, 0.53% and 0% (none) of patients, respectively. In terms of aggressiveness, *ATM* loss was identified by Neeb *et al.* (7) in 68/631 (11%) men with advanced PC; it was associated with increased genomic instability, but not with worse outcomes. For *BRCA1* mutation, interim results from IMPACT (8) did not detect any significant difference in age or tumor characteristics between carriers and noncarriers.

1.3.4, 1.3.5 and 1.3.6: For high-risk localized PC, the prevalence of germline DNA repair gene mutations was 6% in the TCGA cohort (4). In a study of 1,211 men with PC receiving active surveillance (9), DNA repair gene mutations were associated with grade reclassification: 11/26 patients with mutations vs. 278/1,185 without mutations experienced grade reclassification (adjusted hazard ratio = 1.96, *p* = 0.04). While these results show that there are potential benefits of germline testing in these patients, our panel felt that they are outweighed by the potential manifold impacts on patients’ family members, and by resource limitations.

References:

1. Ooi CJ, Fock KM, Makharia GK, Goh KL, Ling KL, Hilmi I, et al. The Asia-Pacific Consensus on Ulcerative Colitis. *J Gastroenterol Hepatol* (2010) 25(3):453-68. Epub 2010/04/08. doi: 10.1111/j.1440-1746.2010.06241.x.

2. Giri VN, Knudsen KE, Kelly WK, Cheng HH, Cooney KA, Cookson MS, et al. Implementation of Germline Testing for Prostate Cancer: Philadelphia Prostate Cancer Consensus Conference 2019. *J Clin Oncol* (2020) 38(24):2798-811. Epub 2020/06/10. doi: 10.1200/JCO.20.00046.

3. Cui M, Gao XS, Gu X, Guo W, Li X, Ma M, et al. Brca2 Mutations Should Be Screened Early and Routinely as Markers of Poor Prognosis: Evidence from 8,988 Patients with Prostate Cancer. *Oncotarget* (2017) 8(25):40222-32. Epub 2017/04/15. doi: 10.18632/oncotarget.16712.

4. Pritchard CC, Mateo J, Walsh MF, De Sarkar N, Abida W, Beltran H, et al. Inherited DNA-Repair Gene Mutations in Men with Metastatic Prostate Cancer. *N Engl J Med* (2016) 375(5):443-53. Epub 2016/07/20. doi: 10.1056/NEJMoa1603144.

5. Castro E, Goh C, Olmos D, Saunders E, Leongamornlert D, Tymrakiewicz M, et al. Germline Brca Mutations Are Associated with Higher Risk of Nodal Involvement, Distant Metastasis, and Poor Survival Outcomes in Prostate Cancer. *J Clin Oncol* (2013) 31(14):1748-57. Epub 2013/04/10. doi: 10.1200/JCO.2012.43.1882.

6. Dong B, Yang B, Li Y, Chen W, Li J, Xu Z, et al. Insights into Chinese Prostate Cancer Germline Gene Mutation Profile: Hoxb13 G84e Mutation Is Unsuitable for Genetic Testing. *J Clin Oncol* (2020) 38(15_suppl):e17515-e. doi: 10.1200/JCO.2020.38.15_suppl.e17515.

7. Neeb A, Herranz N, Arce-Gallego S, Miranda S, Buroni L, Yuan W, et al. Advanced Prostate Cancer with Atm Loss: Parp and Atr Inhibitors. *Eur Urol* (2021) 79(2):200-11. Epub 2020/11/13. doi: 10.1016/j.eururo.2020.10.029.

8. Page EC, Bancroft EK, Brook MN, Assel M, Hassan Al Battat M, Thomas S, et al. Interim Results from the Impact Study: Evidence for Prostate-Specific Antigen Screening in Brca2 Mutation Carriers. *Eur Urol* (2019) 76(6):831-42. Epub 2019/09/21. doi: 10.1016/j.eururo.2019.08.019.

9. Carter HB, Helfand B, Mamawala M, Wu Y, Landis P, Yu H, et al. Germline Mutations in Atm and Brca1/2 Are Associated with Grade Reclassification in Men on Active Surveillance for Prostate Cancer. *Eur Urol* (2019) 75(5):743-9. Epub 2018/10/13. doi: 10.1016/j.eururo.2018.09.021.
